# The Notification of Births Bill

**Published:** 1907-07-06

**Authors:** 


### The Hospital

A Journal of

The Medical Sciences and Hospital Administration.

New Series. No. 15, Vol. I. [No. 1087, Vol. XLII.] SATURDAY, JULY 6, 1907.

### The Notification of Births Bill.

The Notification of Births Bill, though it will probably not become law during the present Session, has reached a stage which shows that its principles commend themselves to the present House of Commons. We have nothing to say here on the general motives and objects of the Bill, and, indeed, for our present argument, we are willing to allow that these are in the highest degree excellent. But it is necessary to observe that certain provisions of the Bill touch the duties and responsibilities of the medical profession very closely, and we venture to say that these provisions are not only unjust to the profession, but also in the highest degree inexpedient in the public interest. It may be well and wise that every birth shall be formally notified to the authorities within thirty-six hours of its occurrence. But we claim that such notification is not, and ought not to be made, the duty of the medical profession. As it stands at present, the Bill demands that the father of the child, if he is residing in the house at the time of birth, shall within thirty-six hours give notice of the event in writing to the local authorities concerned. But exactly the same duty is imposed "upon any person in attendance upon the mother at the time or within six hours after the birth." And this in practice will in a great many instances mean the medical practitioner in charge of the case. This conclusion is confirmed by what has happened under the Notification of Infectious Diseases Act. There, while both the doctor and the head of the house have an equal liability, it has come about that the duty is left entirely to the doctor. And this experience will doubtless repeat itself should the Bill here discussed become law.

Now it is necessary to state clearly what are the objections entertained by the medical profession to the proposed procedure. The most radical and important of these is, that what the Bill demands is an invasion of the confidence which exists, and which it is in the public interest should exist, between a medical practitioner and his patient. The proposal is nothing less than an attempt to make the practitioner, who is called as the private adviser of the patient, an agent and officer of the law. He is engaged, and receives his fee, on behalf of the interests of an individual citizen, yet he is to be compelled by Parliament to adopt and discharge a public function. We hold this to be an unfair demand and a vicious policy. It is in the best interests of the community that the citizens shall regard the confidences they place in their medical advisers as utterly sacred and secure. Whatever undermines this trust is prejudicial to medical practice, and is injurious to the public interest. Hence a proposal which compels a medical practitioner to hasten from his patient's bedside to inform a public official of what has transpired in the sick-room, is to be vigorously condemned and firmly resisted. We are not, let it be noted, contending that the State should not receive this information. That is as it may be. Our claim is that it has no right to force the medical practitioner, the patient's private adviser, to be the informant. And this, first because it compels a breach of professional confidence and so weakens the sanctity of the relations between doctor and patient, and, secondly, because it places upon a private practitioner, public activities which are not part of, and may be inconsistent with, his duty to his patient.

To what has just been said it may be objected that the profession already discharges under the Notification of Infectious Diseases Act similar functions to those demanded in the new Bill. This is perfectly true, but these functions are discharged under the compulsion of the law. The medical profession has now no choice in the matter. Some of us raised objection when the proposal was first made, and have continued the protest as opportunity has offered. Possibly, if a determined opposition had been offered when compulsory notification was first suggested the policy would not have been adopted, and the present natural development of it would not have taken place. We have just the same objections to the direct notification of infectious diseases by the private medical adviser of the patient as we have to the notification of births by the same agent. Both the one and the other involve a failure to recognise and respect a relationship which should be treated as sacred. But because we are already compelled by law to injure this relationship in one set of circumstances, that is no reason why we should quietly acquiesce in a still further invasion. There is an additional point of interest, and those who accepted, more or less readily, the first demand may note its significance. When members of the profession some years ago were called upon to reveal to public officials information which they had gained in their capacity as private medical advisers, the law acknowledged the proceeding by the payment of a modest fee. Now a similar demand is advanced in regard to the notification of births and no such financial recognition is suggested. Such is the natural consequence of departure from principle. The fee, however, is but a trivial comment. Fee or no fee, we hold the provisions of the Bill now before Parliament in so far as they compel notification of births by private medical practitioners to be definitely opposed to the public interest.

